# A Panel Data Analysis of Subjective Well-Being Based on Microblog User Information

**DOI:** 10.3390/healthcare10112305

**Published:** 2022-11-17

**Authors:** Shuijin Li, Tingshao Zhu

**Affiliations:** 1 Department of Psychology, University of Chinese Academy of Sciences, Beijing 100049, China; 2 Institute of Psychology, Chinese Academy of Sciences, Beijing 100101, China

**Keywords:** subjective well-being, China’s Livelihood Index, Granger causality test, fixed-effects model, Easterlin Paradox

## Abstract

Big data modelling using microblogs is applied to acquire nationwide representative panel data on subjective well-being. The analysis directly validates the influence of China’s Livelihood Index on subjective well-being. Using panel data on subjective well-being collected for the period from 2010 to 2021 from users of the Weibo (Sina Corporation, Beijing, China) microblogging platform, this study finds Granger causality running from China’s Livelihood Index to subjective well-being and that the two are positively correlated. We also find Granger causality running from a life stress indicator to a life satisfaction indicator. The education indicator model is found to be positively correlated with life satisfaction and positive emotions, whereas the life stress indicator and life satisfaction are negatively correlated. Medical and health indicators are positively related to life satisfaction, while a negative correlation is found between the traffic indicator model and life satisfaction. The relationship between economic development and subjective well-being also displays bidirectional Granger causality and a positive correlation. However, in China’s provinces and prefecture-level cities with relatively strong economic growth, the correlation between the livelihood index and economic development appears to be weaker. We suggest boosting gross domestic product per capita and absolute per capita income to increase subjective well-being in less developed western China. Bridging the gross domestic product per capita gap nationwide may also positively influence subjective well-being. To achieve this, we suggest measures that include improving medical and health services, alleviating traffic congestion, increasing the teacher–student ratio and improving the education universalisation rate. These steps would improve the equitable and balanced development of China’s Livelihood Index across the country’s 31 provinces.

## 1. Introduction

In the 4th century BC, Aristotle argued that well-being is the ultimate goal of human existence [1,2]. In recent years, some countries have recognised that economic indicators such as gross domestic product (GDP) and gross national product [3] are unable to fully capture the status of a society’s state of development [4]; therefore, an increasing number are turning to the concept of gross national well-being as a basic national statistical indicator [2,5,6,7]. Many Western countries consider the well-being index to be a fundamental indicator of national well-being [2,8,9], and some governments have stated that their objective is to achieve the ‘greatest well-being’ in their countries [7,10]. China also considers well-being to be an important development objective and has stressed the need to facilitate people’s well-being by ensuring they are fulfilled, secure and sustainable [11].

Since the onset of its modern economic reforms China’s economy has grown rapidly, and the Chinese people are moving from finding satisfaction through material things to expressing an increasing desire for personal development [12]. Gongcheng [13] proposed that the goal of livelihood-related activities is grounded in making people happy. To protect and improve people’s well-being, priority should be given to pursuing interests that allow them to achieve a better life [11]. Basic survival needs are met by public services, such as public medical care, social security, environmental protection and infrastructure, and needs for personal development are met through employment, basic education and public cultural activities [2]. Performance indicators based on GDP are being complemented or even replaced by indicators that assess well-being [14], and public services are placing greater emphasis on the importance of people-oriented inheritance and development. From this perspective, countries should implement public services to improve people’s well-being with the goal of establishing a modern service-oriented government [15].

China’s Livelihood Index was developed from research conducted at the Development Research Centre of the State Council [16,17,18,19]. This index consists of two parts: an objective livelihood index and a subjective livelihood index. The subjective component is compiled from the results of an evaluation of the state of livelihood services conducted by telephone or via household surveys. The objective component includes four primary indicators: residents’ quality of life, ecology, the social environment and public services. This reflects the level of development and improvement in people’s livelihood in a given province [17].

Previous studies have examined the interplay between the livelihood index and well-being at a macro-social level. Most of the research on the livelihood index focuses on public services, examining the mechanisms that influence public services and well-being. While well-being has been considered a feasible goal in public policy evaluation [3], the quality of government services is seen as a prerequisite for improving people’s overall well-being. Samanni and Holmberg [4] conclude that the quality of livelihood services, which may be defined as social and public services such as education and health services that are provided by the state, has a remarkable influence on well-being. Zhou et al. [17] report that people’s satisfaction with public services can lead to a substantial increase in their sense of well-being. Zhu et al. [20] suggest that public systems have a direct influence on well-being. Huang and Fu [21] conclude there is an inverted U-shaped relationship between the amount a government spends on livelihood services and people’s well-being. Other studies show that higher expenditures on education increase people’s well-being [22], and public expenditures have been found to have a major influence on the well-being of Americans [23]. Other studies show that people report greater well-being as public expenditures on livelihood services increase [24], and that improvements in public service quality can elevate people’s well-being; for example, satisfaction with basic housing influences well-being far more than three other types of public services [25]. An increase in the employment rate [26] and social welfare policies, such as unemployment benefits, are positively associated with well-being [3]. Bates [5] shows that the culture of countries with individualism has higher happiness than that of countries with collectivism, the subjective happiness of religious people is higher than that of non-religious people and the higher the degree of religious belief, the higher the subjective happiness of people.

However, previous findings are inconsistent and cross-sectional data suffers from a lack of continuity and stability. According to the literature, the factors that influence well-being are all correlated, yet it is difficult to obtain longitudinal results, and causality cannot be determined. Using panel data on well-being collected across 11 consecutive years, we analyse longitudinal and causal relationships between China’s Livelihood Index and people’s well-being. Our work provides a basis for the government to boost well-being by improving policies that aim to improve people’s livelihood.

Zhou et al. [17] use longitudinal data collected from the China Livelihood Index survey from 2010 to 2014. The objective component was used as the livelihood index, and the subjective survey was used to measure residents’ well-being. Their results show no positive correlation between residents’ well-being and the livelihood index in 2010; from 2011 to 2014, there were opposite results for urban versus rural residents in terms of the objective indicators and well-being across four types of public services (compulsory education, medical and health care, ecological environment and social security). The research expenditure on education had a negative relationship with residents’ well-being. Huang and Fu [21] use a life satisfaction metric from the China General Social Survey (CGSS) for the period 2010 to 2015 as a measure of residents’ well-being and data on public service expenditures from the China Statistical Yearbook. They find a significant inverted U-shaped relationship between government expenditure on public services and residents’ well-being. Xu, X.F [27] uses life satisfaction as a measure of national well-being using panel data collected during 2010 to 2016 from the China Family Panel Studies (CFPS) and public service fiscal expenditures from the China Statistical Yearbook to show that government expenditure on livelihood substantially improved people’s well-being. The conclusions drawn from the CFPS data collected from 2010 to 2016 differ from those drawn from the CGSS data collected from 2010 to 2015, as well as from China’s Livelihood Index Survey Group data. In sum, time series data from different sources produce varying results. With the ongoing improvement of public services, more robust longitudinal data are needed for empirical analysis. Therefore, we established the following research hypothesis:

**Hypothesis** **1** **(H1).** *Using time series data, China’s Livelihood Index maintains a long-term positive relationship with well-being, and a causal relationship exists*.

When studying how China’s system of livelihood services influences people’s well-being, the impact of economic development cannot be ignored. However, the relationship between economic growth and well-being has been long debated. The main arguments, which hold that economic growth and well-being are not always positively associated, are the Easterlin Paradox [28] and the well-being paradox [29,30]. Some studies show that the growth rate of GDP per capita and well-being are positively correlated in short-term time series data but not over the long term [31]. Based on cross-sectional data collected from 2003–2013, the wealth of an individual and his country is positively correlated with well-being in the short term; however, GDP per capita and well-being are not significantly correlated [30]. Some studies use individual income as an indicator of economic development. Data collected from 2003 to 2013 reveal that income growth increases women’s subjective well-being, but there is a U-shaped change in that pattern beyond a certain threshold [32].

Studies using cross-sectional data show that well-being is positively correlated with absolute income [31]. Using CGSS data from 2011, one study found that increased income has a positive bearing on well-being [2]. Based on CGSS data collected from six provinces in 2017, residents’ income and regional affluence significantly positively influence subjective well-being [29]. Finally, using data from the World Values Survey collected in 2001, 2007 and 2012, relative income is shown to significantly boost subjective well-being [33]. In addition, a causal relationship between economic growth and well-being has been corroborated through meta-analysis of data collected from 2001–2009 that shows economic growth Granger causes increased well-being [34]. An analysis also shows a causal relationship between increased income and adult mental health [35].

Based on the conclusions in the reviewed literature, we use panel data on subjective well-being to further explore the causal relationship between economic development and well-being. To do so, we established the following hypothesis:

**Hypothesis** **2** **(H2).** *In time series data, there is a causal and positive relationship between economic development and well-being*.

## 2. Materials and Methods

### 2.1. Variables and Data Sources

In the economics of happiness, subjective well-being is a commonly used indicator of well-being. Subjective well-being is a comprehensive evaluation of the quality of life and contains three dimensions: life satisfaction, positive emotions and negative emotions [36,37,38].

In this study, subjective well-being is chosen as the dependent variable. Our source of subjective well-being data is not a direct method based on traditional psychometric measures; instead, we employ psychological modelling based on social media data, using psychometric measures as calibration to obtain subjective well-being. Su et al. [39] argued that psychological modelling has advantages such as timeliness, retrospective measurement and good ecological validity compared to the self-reported method. Ang et al. [40] and Liu [41] establish a calculation model of subjective well-being; specifically, they construct a big data model based on the calibration validity of the Positive And Negative Affect Scale (PANAS) and Satisfaction With Life Scale (SWLS) scales of psychological well-being. Su et al. [39] test the retest reliability of the psychological model. They find the retest interval of the life satisfaction identification model to be one month, with a retest reliability of 0.84. Su et al. [39] indicate that the psychological modelling method exhibits high stability and consistency. The subjective well-being model in this study uses data from a large number of active users of Weibo, a popular microblogging platform made by Sina Corporation in China, to construct province-level clustering. The clustering method used is described in Zhang and Yu [42], Huang et al. [43] and Li et al. [44]. We apply a machine learning model [40,41] to identify month-by-month of subjective well-being in active Weibo users in different geographical areas, which allows us to characterize the subjective well-being of Internet users in multiple geographic regions over different periods of time. The above-mentioned big data model was used to obtain panel data for a total of 132 months, covering 644,243 active Weibo users. Data from the big data model were entered into the subjective well-being model to obtain users’ subjective well-being from January 2010 to December 2021.The demographic data of participants are shown in Table 1.

The objective China’s Livelihood Index was used as a measure of livelihood, i.e., the independent variable, which was calculated using the livelihood index system [45,46,47,48,49]. The livelihood development of each province, city and region was measured according to the measurement method of the updated livelihood index system. The new livelihood index system measurement method primarily examines the lives of residents, public services and the living environment; each element is assessed using macro-level indicator data, including input and expenditure indicators, to measure the index level for the year. The data of objective indicators were obtained from the publicly available Statistical Yearbook of China, which is published by the National Bureau of Statistics of China. Because these indicators are presented in different units, dimensionless processing was applied and equal weights were used to obtain each livelihood index representing the level of livelihood development [7,45,46,47,48,49].

The macro-level indicators acquired from the National Bureau of Statistics of China [50] include the following: GDP per capita, local government expenditure on education, and teacher–student ratio in primary schools, etc. These main control variables are all macro-indicators and were obtained from the National Bureau of Statistics, respectively, by provincial year [7,45,46,47,48,49]. The descriptive statistics for key variables are shown in Table 2.

Determine the individual indicators and calculation formulas of the livelihood index model based on previous literature references [7,45,46,47,48,49,50,51]. The level of economic development was replaced by GDP per capita in Model 1a. Income and spending were obtained by subtracting the per capita consumer spending of all residents from the absolute per capita disposable income of all residents, which was coded in Model 2a.

Two indicators were selected for the education model (Model 4a); one was the proportion of local government expenditure on education, and the other was the average teacher–student ratio, which was defined as the mean of the student–teacher ratio in primary schools, the student–teacher ratio in middle schools, the student–teacher ratio in high schools and the student–teacher ratio in colleges and universities.

The traffic model (Model 5a) was composed of three indicators: traffic congestion, which was calculated by dividing the number of buses per 10,000 people by the number of passengers using buses (per 10,000 passenger-times); urban road space per 10,000 people; and the proportion of local government expenditure on transportation.

The living environment model (Model 6a) used two indicators: the proportion of government expenditure on environmental protection and the green space per 10,000 people [52].

The social security and employment model (Model 7a) consisted of two indicators, as follows: the proportion of government expenditure and the employment indicators. The proportion of expenditure on social security and employment, which was obtained by dividing the local amount of government expenditure on social security and employment by the regular budget expenditure amount. There were two employment indicators, which were the urban unemployment rate and the employment indicator; among them, the employment indicator was calculated using a formula based on the reference literature [45,46,47,48,49]. Spaces with missing data of the number of self-employed people and employees in private units in urban areas in 2020 or the number of urban participants with basic health insurance at the end of the year in 2010 were filled with zeros.

The medical and health care model (Model 8a) was composed of the proportion of government expenditure on medical and health and the indicator of medical matching services. The medical service package used two indicators, doctors and beds, where doctors refers to the number of licensed (assistant) physicians per 10,000 people and beds refers to the number of beds in medical institutions per 10,000 people.

The life stress model (Model 9a) has only one indicator, and its value is the absolute per capita disposable income of urban residents divided by the average sales price of residential commercial properties.

The China livelihood service model (Model 10a) is an integrated model that consisted of the above-mentioned models and is derived from the above-mentioned models by taking equal weights.

The relationships between model names, dependent variables and control variables are shown in Table 3.

### 2.2. Models and Methods

Given that the data source was short panel data, within which T was 11 and N was 31, the panel data models adopted were mainly fixed-effects models and the Granger causality test. Panel data regression was used to quantify the interaction between subjective well-being and economic development or subjective well-being and China’s Livelihood Index. Accordingly, the Granger causality test [53] was used to investigate the causal relationship between subjective well-being and economic development or subjective well-being and China’s Livelihood Index.

According to our literature review, short panel data were applied to conduct the Granger causality test at time periods of 13 [54], 12 [55] and 14 [56] years. In the present study, the short panel data with T = 11 years were also subjected to the Granger causality test.

The panel Granger causality test is shown below (using Equations (1) and (2)):

(1) $$\ln\left(Y_{it}\right)=\gamma +\overset{p}{\underset{m=1}{\sum }}\alpha_{m}\ln\left(Y_{i,t-m}\right)+\overset{p}{\underset{m=1}{\sum }}\beta_{m}\ln\left(X_{i,t-m}\right)$$

(2) $$\ln\left(X_{it}\right)=\delta +\overset{p}{\underset{m=1}{\sum }}\alpha_{m}\ln\left(Y_{i,t-m}\right)+\overset{p}{\underset{m=1}{\sum }}\beta_{m}\ln\left(X_{i,t-m}\right)$$

where *Y* is subjective well-being and *X* represents macro-level indicators, such as economic development level or China’s Livelihood Index; *i* is the number of individual provinces and cities (1–31 in this study); *t* represents the time period, which was 1–11 years; and lag order p was determined according to the Akaike’s information criterion and Schwarz criterion. Lag order is theoretically the minimum value of the Akaike’s information criterion and Schwarz criterion; however, the larger the lag, the greater the data loss. When the order is 3, the lag order will continue to increase with a low degree of variation in Akaike’s information criterion and Schwarz criterion values; the *p* value was determined as 3 in this study.

In Equation (1), the original hypothesis tested was “H_0_: *β*_1_ = *β*_2_ = … = *β*_m_ = 0,” i.e., X does not Granger-cause Y. In Equation (2), the original hypothesis tested was “H_0_: *α*_1_ = *α*_2_ = … = *α*_m_ = 0,” i.e., *Y* does not Granger-cause *X*.

The fixed-effects model was used to examine whether the control variables had a positive or negative effect on subjective well-being and to discern the extent of any effect.

The panel data regression models were as follows (in Equations (3) and (4)):

(3) $$\ln\left(Y_{it}\right)=\alpha_{i}\ln\left(X_{it}\right)=u_{i}=\varepsilon_{it}$$

(4) $$\ln\left(X_{it}\right)=\beta_{i}\ln\left(Y_{it}\right)=u_{i}=\varepsilon_{it}$$

where *μ* is an intercept reflecting individual heterogeneity and *ε* is a perturbation that varies over time and across individuals; *α* is the “*Y–X*” coefficient and describes how subjective well-being varies with the control variable, i.e., when the control variable changes by 1%, people’s well-being changes by *α*%; and *β* is the “*X–Y*” coefficient, which was used to describe how the control variable varies with subjective well-being, i.e., when subjective well-being changes by 1%, the control variable changes by *β*%.

## 3. Results

### 3.1. Panel Unit Root Test

Regression analysis and its correlation tests are only credible when the data are stationary [57]. If time series data show a consistent trend (non-stationary), spurious or pseudo-regressions will arise [57]. To prevent spurious regressions resulting from a common time trend, a panel unit root test was conducted for all indicator data in this study. The Augmented Dickey–Fuller (ADF) test was commonly applied to test the stationarity of each indicator series. The calculation tool was RStudio 2022.02.2+485 (RStudio, Boston, MA, USA).

The ADF test for the panel unit root test resulted in *p* = 0.01 for all variables, with *p* < 0.05 indicating a stationary series. Pseudo-regression was therefore deemed unlikely to occur, and the data were thus believed suited to Granger causality tests, panel data regression analyses and correlation analyses.

Another way to check the stationarity of a time series is to apply the autocorrelation function (ACF) test and partial autocorrelation coefficient (PACF) test, in which the autocorrelation function indicates the correlation between the series and its lag series. Based on the ACF test results, the ACF graphs of three time series all had trailing tails, and, with an increasing K-order, the autocorrelation coefficient decreased and trended toward 0, which indicates a stationary series. From the results shown on the PACF graphs, the cutoff orders of the time series were either 1 or 2, which suggested that the three series were stationary after the differenced orders had been checked.

### 3.2. Panel Data Regression Analysis

RStudio 2022.02.2+485 was applied and combined with data regression methods to investigate the influence of each model of the China’s Livelihood Index on the three dimensions of subjective well-being. First, the F-test, Hausman test and co-integration test were applied to the panel data, and regression analysis was conducted according to the results.

According to the test results, as shown in Table 4, the *p* values of all three tests (F-test, Hausman test and co-integration test) were <0.05. Combining the above three test results, the GDP per capita, per capita disposable income, the education model, traffic model, medical and health model, life stress model and China livelihood service model all passed the three tests.

The fixed model results of the above-mentioned models (Table 5, Table 6 and Table 7) show that GDP per capita was significantly and positively correlated with life satisfaction, absolute per capita disposable income was significantly and positively correlated with life satisfaction, education indicators were significantly and positively correlated with life satisfaction, transportation indicators were negatively correlated with life satisfaction, the medical and health model was significantly and positively correlated with life satisfaction and life stress indicators were significantly and negatively correlated with life satisfaction. Additionally, the interaction term of GDP per capita and the life stress model was positive, which suggests that better regional economic development was negatively correlated with life stress, but marginal values were not achieved. The relationship between the China livelihood service model and life satisfaction was positive, while the traffic model indicators and life stress indicators were negatively correlated with life satisfaction; additionally, the residential environment model, medical and health indicators and life satisfaction were positively correlated. The interaction term of GDP per capita with the education model, traffic model, residential environment model, social security and employment model, medical and health model and life stress model was positive; this indicates that by uplifting the macro-level indicator of this model, there was a positive interaction effect of life satisfaction.

It is noteworthy that GDP per capita changed from being positive in Model 1A to negative in Model 10A; however, this result was not significant, which indicates that, in regions with weaker economic development, an increased value of China’s Livelihood Index still has a positive influence on life satisfaction. Elsewhere, in regions with better economic development, improvements to China’s livelihood services have a weaker bearing on livelihood, which highlights the importance of the equalization of China’s livelihood services. Nevertheless, the interaction term of GDP per capita and the livelihood service model was positive. Due to the disparity between fiscal income and economic development, the central government is expected to devote more funds to developing regions.

As shown in Table 8, the models verified by the three tests included GDP per capita and the education model, residential environment model, medical and health model, life stress model and livelihood service model.

For those models that passed all three tests, the significant model results of the fixed model are shown in Table 9 and Table 10. The results revealed that GDP per capita was significantly positively correlated with positive emotions, while the life stress model was negatively correlated with positive emotions. Likewise, the results concerning negative emotions and the livelihood index model were as follows: the models that passed the three tests were the absolute per capita disposable income and education model. However, the fixed model results of negative emotions and absolute per capita disposable income as well as negative emotions and the education model were not significant.

After integrating the above dimensions of subjective well-being and the fixed model results of life satisfaction, positive emotions, negative emotions and each livelihood index in the time series, the findings are as follows. First, traffic congestion and life satisfaction were negatively correlated. Additionally, the proportion of investment in traffic and life satisfaction were negatively correlated, urban road space per 10,000 people was negatively correlated with life satisfaction, the medical and health index of the number of licensed physicians were positively correlated with life satisfaction, and the life stress index was negatively correlated with life satisfaction. Finally, the education index was positively correlated with life satisfaction, while the proportion of investment in education was negatively correlated with life satisfaction.

It was also determined that the overall model of livelihood service was significantly positively correlated with life satisfaction, the traffic model and life satisfaction were negatively correlated, the correlation between the medical and health model and life satisfaction was positive, living environment was positively related to life satisfaction and life stress was negatively correlated with positive emotions. Therefore, according to the above results, the hypothesis that livelihood service is positively correlated with subjective well-being (H2) was verified in the time series data.

### 3.3. Granger Causality Analysis

After regression analysis was conducted, it was necessary to further verify the causal relationship between China’s Livelihood Index model and subjective well-being. For this purpose, the Granger causality test could be carried out for those that passed co-integration tests. As shown in Table 5, Table 6, Table 7, Table 9 and Table 10, due to the non-significant results of negative emotions and livelihood index indicators in the regression analysis, an analysis of Granger causality was not performed. The Granger causality analysis was only performed for those models that had significant regression results and passed the co-integration test.

The results of the co-integration test, shown in Table 11, were as follows: in the ADF test for residuals of all variables, it was determined that *p* < 0.05, which indicates the existence of a long-term stable equilibrium. In addition, GDP per capita, absolute per capita disposable income of all residents, the life stress model and the livelihood service model all passed the co-integration test for life satisfaction.

After conducting the co-integration test (Table 12), we found that the ADF test for residuals of all variables resulted in *p* < 0.05, which indicated a long-term stable equilibrium. GDP per capita, the absolute per capita medical and health model and the livelihood service model all passed the co-integration test for positive emotions.

The RStudio 2022.02.2+485 Granger causality test was employed to obtain the following results, which are also shown in Table 13. Bidirectional Granger causality was found for the effect of GDP per capita on life satisfaction and positive emotions of subjective well-being. The Granger causality relationship between absolute per capita disposable income and life satisfaction of subjective well-being was bidirectional in the time series of H2, and a causal relationship between economic development and subjective well-being was confirmed. Bidirectional Granger causality also ran from the education model to life satisfaction, while unidirectional causality ran from the life stress model to life satisfaction and the livelihood service model to life satisfaction. In the time series of H1, we confirmed that a degree of causality existed between economic development and subjective well-being.

## 4. Discussion

Much attention has been paid to people’s well-being in the context of implementing policies that affect livelihood services. According to Pigou’s theory of welfare economics, people expect to share the fruits of economic development [2]. We calculated Weibo users’ well-being using big data models and empirically analysed the bidirectional influence of subjective well-being and the livelihood index. Applying Granger causality tests and a fixed model analysis revealed the following results. First, economic development is positively correlated with the well-being of people’s livelihood in the time series, which is consistent with the results of a cross-sectional study by Chong et al. [31] that shows well-being is positively correlated with absolute income level. Economic development is found to Granger-cause people’s livelihood well-being, where per capita GDP is taken as the observed measure of economic growth, which is consistent with Cai et al. [34], who show that economic growth Granger-causes an increase in well-being. We also used per capita disposable income as an indicator of economic development, confirming that economic growth Granger-causes an increase in well-being. This result is consistent with Thomson [35], who shows that income has a causal relationship with mental health in adults. The above conclusions support H2, which posits a causal and positively correlated relationship between economic development and subjective well-being in the time series.

Next, we identified the mechanism underlying the links between various livelihood index values on well-being; in the time series, education indicators were positively correlated with well-being, a result that is similar to those found in other studies [22]. In addition, education indicators are found to Granger-cause life satisfaction. Among them, the teacher–student ratio is positively associated with life satisfaction, a finding that is consistent with the positive directionality in Zhang, H. [2], which shows that when the basic education factor (primary teacher–student ratio or middle school teacher–student ratio) increases by one unit, the probability of a higher level of well-being increases by 12.2%, and the teacher–student ratio is positively correlated with well-being. Traffic congestion indicators are negatively correlated to well-being, as traffic congestion increases commuting time, resulting in a decrease in well-being. This finding is consistent with Wu, J.J [58], who uses cross-sectional data from the 2010 CFPS to show that commute time has a negative impact on personal well-being and life satisfaction. We find that increasing the ratio of health care workers to patients in health care indicators enhances well-being, which is consistent with Chu et al. [59], who find health insurance has a significant positive effect on subjective well-being. The life stress indicator used in this study is per capita disposable income for urban residents divided by the average price of residential housing. We show that this life stress indicator is negatively related to life satisfaction, consistent with Li et al. [60] and Yuan, J.X. [61]. Li et al. [60] point out that an increase in the house price-to-income ratio implies a decrease in individual utility, which has a negative effect on the willingness of a mobile population to stay in the same place over the long term. Yuan, J.X. [61] points out that the pressure of high housing prices significantly affects the subjective well-being of a mobile population, and an increase in housing price pressure reduces subjective well-being and individual productivity. In our study, we find the life stress indicator Granger-causes life satisfaction.

This study also found that the per capita GDP and the life stress model by a positive, and in the strong economic development area negative correlation with stress in life, and has not reached the marginal value. The result indirectly shows strong regional economic development and that the range of the per capita disposable income increases the proportion of residential housing prices and that rise far behind the pace of the region.

Based on the relationship between the livelihood index and subjective well-being, we recommend that governments in China develop a transportation system to ease pressure on public transport and reduce traffic, increase student–faculty ratios, increase the ratio of medical staff per patient, improve per capita income, stabilize residential housing prices, improve satisfaction with people’s livelihood and improve people’s overall well-being.

Our results supported H1, as Granger causality runs from the overall indicators of livelihood services to well-being and shows positively correlated. This is consistent with the results of Ma, L. [7] and Xuan et al. [24]. Ma, L. combines the four public services of public education, health care, environmental protection and public transportation into a single indicator of public service performance. He finds that public service performance has a significant positive effect on well-being, and the level of public service has a significant positive correlation with well-being. Xuan et al. divide public services into basic services, public security services, social public services and economic public services, and show that public services have a significant positive impact on residents’ well-being. Sub-indicators of social public services (number of beds in medical institutions, local government expenditure on education per capita, etc.) and basic public services (number of buses per 10,000 people, urban road space per 10,000 people, etc.) all significantly increase residents’ well-being. In this study, we find positive values for the cross-multipliers of GDP per capita with education, traffic, residential environment, social security and employment, medical and health and life stress. These results offer new insights that increasing the indicators for these areas has a positive interaction effect on life satisfaction, especially for the terms involving social security and employment, and increasing the indicators for social security and employment also has a positive effect on subjective well-being.

We also tested the causality between residents’ well-being of subjective well-being and GDP per capita in the opposite direction. The results showed that the effect of residents’ life satisfaction on GDP per capita and the effect of positive emotion on GDP per capita were not excluded. At the same time, the causality of residents’ well-being of subjective well-being on the education model was tested in the opposite direction, and the results showed that the effect of life satisfaction on the education model in the opposite direction was not excluded. This result suggests that when residents’ life satisfaction or positive emotions are stable or sustainable, short-term benefits may result from residents increasing their labour productivity, or long-term effects may be produced by residents increasing their knowledge. In addition, the old-age security component of China’s Livelihood Index protects retirees, the medical security system establishes basic medical care and insurance against serious illness and the medical and health component promotes the health of residents, increasing average life expectancy. This may help to stimulate domestic demand in the short term, which could have an impact on economic growth.

The study has limitations. First, the data of subjective well-being were mainly obtained from social media. However, the situation may be different online and offline. The information from social media does not include data on education level, personal income or marital status.

## 5. Conclusions

This study explores how China’s Livelihood Index influences subjective well-being through an analysis of panel data on subjective well-being, and we build a two-way fixed effects model and a Granger causality model to achieve the purpose of the experiment. Our study shows that there is a unidirectional Granger causality between China’s Livelihood Index and subjective well-being. Besides, there is a positive correlation between China’s Livelihood Index and subjective well-being. Furthermore, there is a bi-directional Granger causality between economic development and subjective well-being, and they are significantly positively correlated. The findings are a complement to China’s ‘Easterlin Paradox’, and this evidence provides the government with a theoretical basis to improve subjective well-being through a suite of policies for improving people’s livelihood in a situation where the level of economic development varies from place to place nationwide. The policy implication of this study is that the government may boost subjective well-being by improving public welfare services and adjusting China’s Livelihood Index when economic development influences subjective well-being to a certain degree.

## Figures and Tables

**Table 1:** Demographics of active microblog users.

	Variables	Frequency	Proportion (%)
Gender	Male	263,990	40.98%
	Female	380,253	59.02%
AGE	18–28	36,690	5.70%
	28–38	85,236	12.80%
	38–48	11,485	1.95%
	48–58	1976	0.34%
	Above 58	621	0.10%
	NA:	508,235	78.89%
geographical position	Beijing	58,227	9.04%
	Tianjin	8595	1.47%
	Hebei	9341	1.62%
	Shanxi	11,160	1.96%
	Inner Mongolia	7384	1.33%
	Liaoning	17,988	3.27%
	Jilin	9803	1.84%
	Heilongjiang	15,872	3.04%
	Shanghai	74,164	14.66%
	Jiangsu	33,641	7.79%
	Zhejiang	35,953	9.03%
	Anhui	12,257	3.38%
	Fujian	23,273	6.65%
	Jiangxi	11,744	3.60%
	Shandong	23,002	7.31%
	Henan	14,796	5.07%
	Hubei	20,602	7.44%
	Hunan	14,967	5.84%
	Guangdong	112,634	46.64%
	Guangxi	18,233	14.15%
	Hainan	3824	3.46%
	Chongqing	9349	8.76%
	Sichuan	26,502	27.20%
	Guizhou	9335	13.16%
	Yunnan	11,670	18.95%
	Xizang	6227	12.47%
	Shaanxi	17,094	39.12%
	Gansu	11,781	44.28%
	Qinghai	1909	12.88%
	Ningxia	6597	51.08%
	Xinjiang	6319	48.92%

**Table 2:** Descriptive statistics for key variables.

	Variable	Mean	SD
SWB	LS	2.455	2.922
	PE	46.7	27.061
	NE	45.922	15.168
ED	GDP-P	51,219.944	26,848.556
IS	DI-P	22,221.179	11,210.827
	CS-P	15,876.739	7231.626
Education	LGEE	787.039	527.576
	SRIPS	16.312	2.366
	SRIMS	12.721	2.176
	SRIHS	13.883	2.514
	SRICU	17.544	1.212
Traffic	NB-P	11.778	4.017
	NPUB	209,814.5	141,975.947
	LGET	290.02	174.783
	UR-P	14.823	5.731
LE	GS-P	12.768	2.868
	LGEP	139.536	98.207
SSE	LGSSE	611.251	384.737
	NUPI	1158.394	971.986
	NUBH	2587.846	971.986
	NEPU	2587.846	2631.25
	NSEPU	541.133	393.344
	UUR	582.28	395.181
MHC	LGMH	371.064	262.203
	NBMI	51.333	11.789
	NALP	23.44	6.613
LSS	DIU-P	30,183.924	11,182.677
	APRC	7401.366	5730.752
	LGGE	4719.64	2831.012

Abbreviations: SWB, Subjective well-being; ED, Economic development; IS, Income and spending; MHC, Medical and health care; SSE, Social security and employment; LS, Life satisfaction; PE, Positive emotions; NE, Negative emotions; GDP-P, GDP per capita; DI-P, per capita disposable income of all residents; CS-P, per capita consumer spending of all residents; DIU-P, per capita disposable income of urban residents; APRC, Average sales price of residential commercial properties; LGEE, Local government expenditure on education; SRIPS, student–teacher ratio in primary schools; SRIMS, student–teacher ratio in middle schools; SRIHS, student–teacher ratio in high schools; SRICU, student–teacher ratio in colleges and universities; LGMH, Local financial expenditure on medical and health care; NBMI, Number of beds in medical institutions; NALP, Number of licensed (assistant) physicians; LGSSE, Local government expenditure on social security and employment; NUPI, Number of urban workers participating with pension insurance; NUBH, Number of urban participants with basic health insurance at the end of the year; NEPU, Number of employed people in urban areas; NSEPU, Number of self-employed people and employees in private units in urban areas; UUR, Urban unemployment rate; GS-P, Green space per 10,000 people; LGEP, Local government expenditure on environmental protection; NB-P, Number of buses per 10,000 people; NPUB, Number of passengers using buses; LGET, Local government expenditure on transportation; UR-P, Urban road space per 10,000 people; LE, Living environment; LSS, Life stress; LGGE, Local government expenditure on General budget.

**Table 3:** The relationships between model names, dependent variables and control variables.

Model-Name	Dependent Variable	Control Variable
GDP-P	LS, PE, NE	GDP-P
ADI-P	LS, PE, NE	DI-P, CS-P
Education	LS, PE, NE	SRIPS, SRIMS, SRIHS, SRICU, LGEE, LGGE
Traffic	LS, PE, NE	TC, UR-P, LGET, LGGE
LE	LS, PE, NE	GS-P, LGEP, LGGE
SSE	LS, PE, NE	LGSSE, NUPI, NUBH, NEPU, NSEPU, UUR, LGGE
MHC	LS, PE, NE	LGMH, NBMI, NALP, LGGE
LSS	LS, PE, NE	DIU-P, APRC, LGGE
CLS	LS, PE, NE	Education, Traffic, LE, SSE, MHC, and LSS model variable

Abbreviations: GDP-P, GDP per capita; LE, Living environment; SSE, Social security and employment; MHC, Medical and health care; LSS, Life stress; CLS, China’s livelihood service; LS, Life satisfaction; PE, Positive emotions; NE, Negative emotions; DI-P, per capita disposable income of all residents; CS-P, per capita consumer spending of all residents; SRIPS, student–teacher ratio in primary schools; SRIMS, student–teacher ratio in middle schools; SRIHS, student–teacher ratio in high schools; SRICU, student–teacher ratio in colleges and universities; LGEE, Local government expenditure on education; LGGE, Local government expenditure on General budget; NB-P, Number of buses per 10,000 people; NPUB, Number of passengers using buses; TC, Traffic Congestion, the value of NB-P divided by NPUB; LGET, Local government expenditure on transportation; UR-P, Urban road space per 10,000 people; GS-P, Green space per 10,000 people; LGEP, Local government expenditure on environmental protection; LGSSE, Local government expenditure on social security and employment; NUPI, Number of urban workers participating with pension insurance; NUBH, Number of urban participants with basic health insurance at the end of the year; NEPU, Number of employed people in urban areas; NSEPU, Number of self-employed people and employees in private units in urban areas; UUR, Urban unemployment rate; DIU-P, per capita disposable income of urban residents; APRC, Average sales price of residential commercial properties; LGMH, Local financial expenditure on medical and health care; NBMI, Number of beds in medical institutions; NALP, Number of licensed (assistant) physicians.

**Table 4:** Test results of life satisfaction and model variables.

Model-Name	Cointegration Test	F-Test	Hausman Test
GDP-P	0.00163	<2.2 × 10^−16^	0.003312
ADI-P	0.000107	<2.2 × 10^−16^	0.00002349
Education: GDP-P	0.004081	<2.2 × 10^−16^	0.02585
Traffic: GDP-P	0.000607	2.2 × 10^−16^	2.2 × 10^−16^
LE: GDP-P	0.0000331	2.2 × 10^−16^	0.4213
SSE: GDP-P	3.86 × 10^−11^	2.2 × 10^−16^	0.9347
MHC: GDP-P	0.0000766	2.2 × 10^−16^	2.2 × 10^−16^
LSS: GDP-P	0.009997	<2.2 × 10^−16^	9.589 × 10^−10^
CLS: GDP-P	0.009997	2.2 × 10^−16^	2.2 × 10^−16^

Abbreviations: GDP-P, GDP per capita; ADI-P, Absolute per capita disposable income of all residents, its value is DI-P minus CS-P; LE, Living environment; SSE, Social security and employment; MHC, Medical and health care; LSS, Life stress; CLS, China’s livelihood service; Education: GDP-P represents the interaction effect of the two. By analogy.

**Table 5:** Fixed Model results of life satisfaction and GDP-P, ADI-P and Education model.

Variable	GDP-P Model	ADI-P Model	Education Model
GDP-P	0.16682 (2.1194 *)		0.370982 (3.4659 ***)
ADI-P		0.135898 (2.19 *)	
SR			0.615926 (4.4652 ***)
PLGEE			−0.542911 (−5.3288 ***)
GDP-P: SR: PLGEE			−0.044024 (−0.7473)

Abbreviations: GDP-P, GDP per capita; DI-P, per capita disposable income of all residents; CS-P, per capita consumer spending of all residents; ADI-P, Absolute per capita disposable income of all residents, its value is DI-P minus CS-P; SRIPS, student–teacher ratio in primary schools; SRIMS, student–teacher ratio in middle schools; SRIHS, student–teacher ratio in high schools; SRICU, student–teacher ratio in colleges and universities; SR, student–teacher ratio, it’s the average of SRIPS, SRIMS, SRIHS, SRICU; LGEE, Local government expenditure on education; LGGE, Local government expenditure on General budget; PLGEE, Proportion of expenditure on education, the value of LGEE divided by LGGE; Data are nondimensionalized. The two-way fixed model controlled for years and provinces. t-values are in brackets. *** *p* < 0.001, * *p* < 0.05. GDP-P:SR:PLGEE represents the interaction effect of the three variables. By analogy.

**Table 6:** Fixed Model results of life satisfaction and Traffic, MHC and LSS model.

Variable	Traffic Mode	MHC Model	LSS Model
GDP-P	0.815907 (9.2313 ***)	0.1315045 (0.6664)	0.407138 (4.5254 ***)
TC	−0.367182 (−2.7186 **)		
PLGET	−0.139210 (−2.3644 *)		
UR-P	−0.157976 (−2.3139 *)		
GDP-P: TC: PLGET: UR-P	0.112629 (1.5563)		
NALP		0.36974500 (2.2295 *)	
NBMI		−0.0007712 (−0.0050)	
PLGMH		0.01250931 (0.1250)	
GDP-P: NALP: NBMI: PLGMH		−0.1492478 (−2.5240)	
IHPR			−0.658125 (−6.3365 ***)
GDP-P: IHPR			0.205750 (3.4285 ***)

Abbreviations: GDP-P, GDP per capita; NB-P, Number of buses per 10,000 people; NPUB, Number of passengers using buses; TC, Traffic Congestion, the value of NB-P divided by NPUB; LGGE, Local government expenditure on General budget; LGET, Local government expenditure on transportation; PLGET, Proportion of expenditure on transportation, the value of LGET divided by LGGE; UR-P, Urban road space per 10,000 people; NALP, Number of licensed (assistant) physicians; NBMI, Number of beds in medical institutions; LGMH, Local financial expenditure on medical and health care; PLGMH, Proportion of expenditure on medical and health care, the value of LGMH divided by LGGE; DIU-P, per capita disposable income of urban residents; APRC, Average sales price of residential commercial properties; IHPR, income house price ratio, the value of DIUP divided by APRC; Data are nondimensionalized. The two-way fixed model controlled for years and provinces. t-values are in brackets. *** *p* < 0.001, ** *p* < 0.01, * *p* < 0.05. GDP-P:TC:PLGET:UR-P represents the interaction effect of the four variables. By analogy.

**Table 7:** Fixed Model results of life satisfaction and CLS model.

Model-Name	CLS Model
GDP-P	−0.1241445 (−1.2101)
Education	−0.1398902 (−2.2636 *)
Traffic	−0.3388001 (−10.5217 ***)
LE	0.1224539 (2.6358 **)
SSE	−0.0070484 (−0.2344)
MHC	0.1601171 (5.5365 ***)
LSS	−0.5168957 (−5.6540 ***)
GDP-P: Education: Traffic: LE: SSE: MHC: LSS	0.0312291 (2.5082 *)

Abbreviations: GDP-P, GDP per capita; LE, Living environment; SSE, Social security and employment; MHC, Medical and health care; LSS, Life stress; CLS, China’s livelihood service; Data are nondimensionalized. The two-way fixed model controlled for years and provinces. t-values are in brackets. *** *p* < 0.001, ** *p* < 0.01, * *p* < 0.05. GDP-P: Education: Traffic: LE: SSE: MHC: LSS represents the interaction between the seven model variables.

**Table 8:** Test results of positive emotions and model variables.

Model-Name	Cointegration Test	F-Test	Hausman Test
GDP-P	4.58 × 10^−11^	0.004124	0.000005455
ADI-P	3.91 × 10^−14^	0.361	0.02249
Education: GDP-P	0.00448	0.00001637	2.335 × 10^−09^
Traffic: GDP-P	0.00000401	0.000404	0.1234
LE: GDP-P	0.0271	0.009663	0.0112
SSE: GDP-P	1.032 × 10^−11^	0.0774	0.1423
MHC: GDP-P	0.000725	0.0001144	6.999 × 10^−10^
LSS: GDP-P	1.16 × 10^−10^	6.887 × 10^−09^	8.439 × 10^−15^
CLS: GDP-P	2.2 × 10^−16^	0.00005847	0.000003716

Abbreviations: GDP-P, GDP per capita; ADI-P, Absolute per capita disposable income of all residents, its value is DI-P minus CS-P; LE, Living environment; SSE, Social security and employment; MHC, Medical and health care; LSS, Life stress; CLS, China’s livelihood service; Education model: GDP-P represents the interaction effect of the two. By analogy.

**Table 9:** Fixed Model results of positive emotions and GDP-P, Education and MCH model.

Variable	GDP-P Model	Education Model	MHC Model
GDP-P	0.724246 (7.5983 ***)	1.057556 (7.9764 ***)	1.232817 (5.1839 ***)
SR		0.822044 (4.8112 ***)	
PLGEE		−0.335524 (−2.6587 **)	
GDP-P: SR: PLGEE		0.040748 (0.5584)	
NALP			0.304616 (1.5241)
NBMI			−0.183112 (−0.9826)
PLGMH			−0.189702 (−1.5731)
GDP-P: NALP: NBMI: PLGMH			−0.285471 (−4.0057 ***)

Abbreviations: GDP-P, GDP per capita; SRIPS, student–teacher ratio in primary schools; SRIMS, student–teacher ratio in middle schools; SRIHS, student–teacher ratio in high schools; SRICU, student–teacher ratio in colleges and universities; SR, student–teacher ratio, it is the average of SRIPS, SRIMS, SRIHS, SRICU; LGEE, Local government expenditure on education; LGGE, Local government expenditure on General budget; PLGEE, Proportion of expenditure on education, the value of LGEE divided by LGGE; NALP, Number of licensed (assistant) physicians; NBMI, Number of beds in medical institutions; LGMH, Local financial expenditure on medical and health care; PLGMH, Proportion of expenditure on medical and health care, the value of LGMH divided by LGGE; Data are nondimensionalized. The two-way fixed model controlled for years and provinces. t-values are in brackets. *** *p* < 0.001, ** *p* < 0.01. GDP-P:SR:PLGEE represents the interaction effect of the three variables. By analogy.

**Table 10:** Fixed Model results of positive emotions and CLS model.

Model-Name	CLS Model
GDP-P	0.510318 (3.3016 **)
Education	0.018225 (0.1957)
Traffic	−0.074277 (−1.5311)
LE	0.092019 (1.3147)
SSE	0.068636 (1.5154)
MHC	0.081536 (1.8713)
LSS	−0.821207 (−5.9623 ***)
GDP-P: Education: Traffic: LE: SSE: MHC: LSS	0.017236 (1.0267)

Abbreviations: GDP-P, GDP per capita; LE, Living environment; SSE, Social security and employment; MHC, Medical and health care; LSS, Life stress; CLS, China’s livelihood service; Data are nondimensionalized. The two-way fixed model controlled for years and provinces. t-values are in brackets. *** *p* < 0.001, ** *p* < 0.01. GDP-P:Education:Traffice:LE:SSE:MHC:LSS represents the interaction between the seven model variables.

**Table 11:** The test results of cointegration between life satisfaction and each model.

Model-Name	ADF Test
GDP-P	0.00163
ADI-P	0.000107
Education: GDP-P	0.0000129
Traffic: GDP-P	0.0775
MHC: GDP-P	0.733
LSS: GDP-P	0.00988
CLS: GDP-P	0.02011

Abbreviations: GDP-P, GDP per capita; ADI-P, Absolute per capita disposable income of all residents, its value is DI-P minus CS-P; MHC, Medical and health care; LSS, Life stress; CLS, China’s livelihood service; Education: GDP-P represents the interaction effect of the two model. By analogy.

**Table 12:** The test results of cointegration between positive emotions and each model.

Model-Name	ADF Test
GDP-P	4.58 × 10^−11^
Education: GDP-P	0.517
MHC: GDP-P	0.000000001
CLS: GDP-P	0.001558

Abbreviations: GDP-P, GDP per capita; MHC, Medical and health care; CLS, China’s livelihood service; Education: GDP-P represents the interaction effect of the two models. By analogy.

**Table 13:** The results of Granger’s causality test.

	F Statistics	Prob.
GDP-P Granger-causes LS	32.139	<2.2 × 10^−16^ ***
LS Granger-causes GDP-P	2.643	0.04928 *
GDP-P Granger-causes PE	29.378	<2.2 × 10^−16^ ***
PE Granger-causes GDP-P	13.524	2.408 × 10^−08^ ***
ADI-P Granger-causes LS	51.088	<2.2 × 10^−16^ ***
LS Granger-causes ADI-P	6.584	0.0002469 ***
Education Granger-causes LS	5.6942	0.0008218 ***
LS Granger-causes Education	5.2206	0.001559 ***
LSS Granger-causes LS	2.7231	0.04434 *
LS Granger-causes LSS	1.8151	0.1442
CLS Granger-causes LS	43.752	2.2 × 10^−16^ ***
LS Granger-causes CLS	2.5297	0.05718

Abbreviations: GDP-P, GDP per capita; LS, Life satisfaction; PE, Positive emotions; ADI-P, Absolute per capita disposable income of all residents, it’s value DI-P minus CS-P; LSS, Life stress; CLS, China’s livelihood service; Data are nondimensionalized. The two-way fixed model controlled for years and provinces. *** *p* < 0.001, * *p* < 0.05.

## Data Availability

The datasets generated for this article are not readily available because the original data is not publicly available. If necessary, we can provide data sets on the three dimensions of subjective well-being. Requests for access to the dataset should be submitted to the appropriate author.
